# Corticosteroids are effective in the treatment of viruspositive post‐COVID myoendocarditis with high autoimmune activity

**DOI:** 10.1002/clc.23978

**Published:** 2023-01-24

**Authors:** Olga Blagova, Lutokhina Yuliya, Polina Savina, Evgeniya Kogan

**Affiliations:** ^1^ The First Sechenov Moscow State Medical University (Sechenov University) Moscow Moskva Russian Federation; ^2^ V.N. Vinogradov Faculty Therapeutic Clinic I.M. Sechenov First Moscow State Medical University (Sechenov University) Moscow Russia; ^3^ Department of Pathology, N.V. Sklifosovsky Institute of Clinical Medicine I.M. Sechenov First Moscow State Medical University (Sechenov University) Moscow Russia

Dear Editor, We read with interest the letter to the editor (Lucas Scárdua Silva, Vinícius Oliveira Boldrini, Ana Maria Marques, Clarissa Lin Yasuda, PhD. Post‐COVID myocarditis with anti‐heart antibodies persistence: clues for autoimmunity?).

The authors of the letter briefly review the publications showing various autoimmune reactions in patients with predominantly acute severe acute respiratory syndrome coronavirus 2 (SARS‐CoV‐2) infection.^1^

We were interested to learn of a study that showed high levels of anti‐heart antibodies in patients with acute COVID‐19.^2^ A limitation of this study is the fact that 2/3 of the comparison group consisted of patients with dilated cardiomyopathy of unknown nature. As dilated cardiomyopathy (DCM) is often inflammatory in nature (inflammatory cardiomyopathy or myocarditis), the high antibody levels found in patients with DCM may reflect the myocarditis. At the same time, in 1/3 of the comparison group with aortic valve disease no increase in anti‐heart antibodies was found, which also seems quite reasonable. This heterogeneity in the comparison group makes the study difficult to interpret. The authors revealed differences in the spectrum of anti‐heart antibodies in patients with COVID‐19 and DCM: immunoglobuline M (IgM) predominated in the first case while IgG—in the second case, which may indicate a chronic inflammatory process in patients with DCM. Anti‐heart antibodies of IgM class in patients with acute COVID‐19 do not yet mean the persistent inflammation in myocardium, follow‐up of such patients is necessary.

In our previous study, anti‐heart antibody levels were examined independently of the presence of cardiac symptoms in acute COVID‐19.^3^ However, only four patients had a history of chronic myocarditis and were treated with immunosuppressive therapy. Their level of anti‐heart antibodies was not significantly higher than normal, which we regard as a result of baseline therapy of myocarditis. At the same time, in patients without a history of myocarditis and cardiac dilatation, anti‐heart antibodies were detected in a high percentage of cases (73.5%) and correlated with the appearance of new cardiac symptoms (in particular, pericardial effusion, and atrial fibrillation). Some types of antibodies correlated also with pneumonia severity, respiratory failure, need for invasive ventilation, chest pain, low QRS voltage, levels of C‐reactive protein, lactate dehydrogenase and prognosis (mortality). These data allowed us to conclude that anti‐heart antibodies can be considered as part of the systemic immune and inflammatory response in COVID‐19. In the long term after coronavirus infection we did not observe the formation of chronic myocarditis in the proportion of patients who were prospectively followed‐up.

Other studies of anti‐heart antibodies in patients with coronavirus infection (both in the acute phase and even more so in the long term) are still unknown to us. Interferon is an interesting marker of the antiviral inflammatory response, but the correlations of its levels with clinical manifestations are not clear.

It seems that a pronounced autoimmune response during acute coronavirus infection is only a potential factor in the development of chronic autoimmune diseases within long‐COVID. There are reports in the literature of systemic autoimmune diseases induced by SARS‐CoV‐2 infection.^4^ However, reports of morphologically verified myocarditis in the long‐term after coronavirus infection (as part of long‐COVID) are still absent. In one of the few studies of probable SARS‐Cov‐2‐induced myocarditis using endomyocardial biopsy and cardiac MRI, the diagnosis of myocarditis could not be morphologically confirmed in any case, despite the presence of all CMR‐Lake Louise criteria in several patients.^5^

However, the role of coronavirus or coronavirus proteins persistence in various organs and tissues in the induction of systemic autoimmune disease is not clear. Our data suggest that coronavirus persists in the myocardium in the majority of patients with morphologically verified post‐COVID myoendocarditis. However, an almost total increase in anti‐heart antibody levels in the same patients indicates the autoimmune nature of myocarditis.^6^ More studies on myocarditis induced by SARS‐Cov‐2 vaccines also support a predominant role of autoimmune reactions in the development of myocarditis.^7^ In particular, autoimmune (including eosinophilic) reactions to the S‐protein of coronavirus, which is a component of most vaccines, are thought to play a leading role in the development of this myocarditis.

As effective antiviral drugs for the treatment of coronavirus infection are still absent and immune reactions clearly predominate, the administration of corticosteroids seems a promising way to treat postvaccinal myocarditis. Below we present the results of a 6‐month complex treatment of 14 patients with morphologically verified myocarditis, which in all patients included the administration of medium‐dose corticosteroids (Table 1).

**Table 1:** Therapy of patients with post‐COVID myoendocarditis.

Medications	*n* (%)
Methylprednisolone, *n*	14 (100%)
Methylprednisolone dose	24−40 mg/day
Mycophenolate mofetil, *n*	1 (7%)
Hydroxychloroquine 200 mg, *n*	1 (7%)
angiotensin converting enzyme inhibitor inhibitors/angiotensin receptor and neprilysin inhibitor	13 (93%)
β‐blockers	12 (86%)
Mineralocorticoid receptor antagonists	8 (57%)
Dapogliflozin	9 (64%)
Anticoagulants	8 (57%)
Amiodarone	5 (36%)

**Figure 1:**
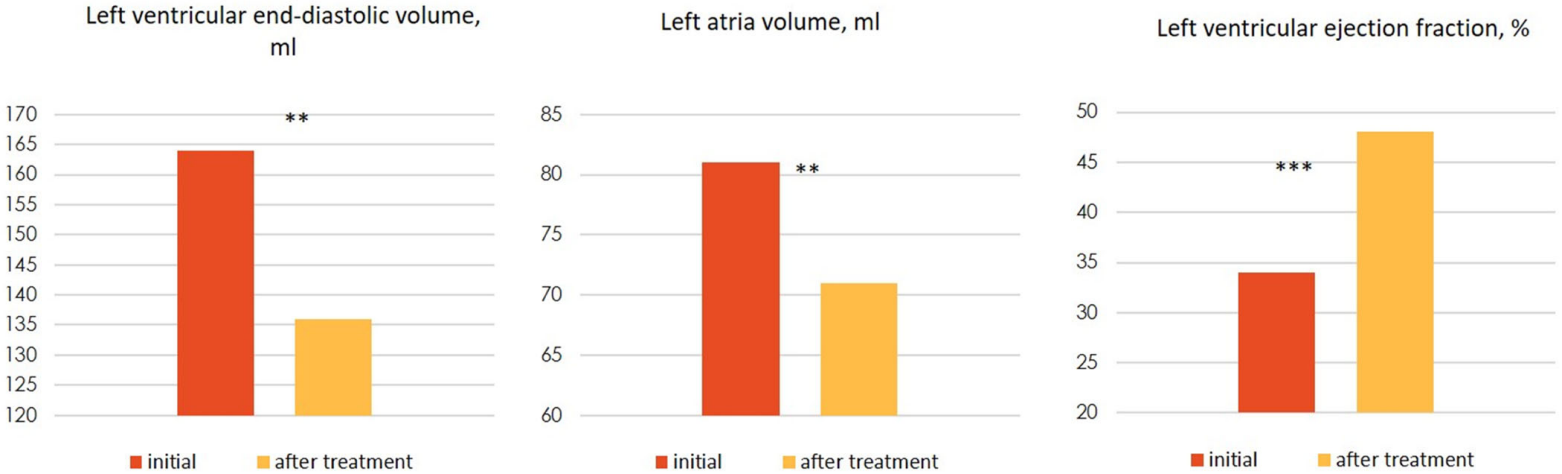
Dynamics of structural and functional parameters of the heart as a result of complex therapy (**p < .01; ***p < .001).

There was a significant increase in left ventricular ejection fraction, a decrease in end‐diastolic volume and left atrial volume (Figure 1), which was accompanied by clinical improvement (reduction in the severity of heart failure) in all patients. In one case (in the absence of coronavirus RNA in the myocardium), mycophenolate mofetil at a dose of 2 g/day was additionally prescribed, and in another case hydroxychloroquine at a minimum dose (200 mg/day) was given. One patient with severe lymphocytic myocarditis died, despite achieving clinical improvement with an increase in ejection fraction of 48%−50%. The cause of death is unknown, but thromboembolic complications (persistent atrial fibrillation and inadequate medication) can be suspected. No progression of myocardial dysfunction was observed in any case, suggesting that corticosteroids are safe in coronavirus‐positive patients. A repeat biopsy in one patient showed absence of SARS‐Cov‐2 RNA in the myocardium and resolution of myocarditis on steroid therapy. Anti‐heart antibody levels decreased significantly in all patients. Maintenance therapy with methylprednisolone 4 mg/day is ongoing, and additional cytostatics should be considered in these patients.
